# Soil-Transmitted Helminths in Kindergarten Children: Prevalence, Intensity and Associated Factors in Bule Hora Town

**DOI:** 10.1155/2024/9356919

**Published:** 2024-09-13

**Authors:** Wako Dedecha, Tibeso Gemechu, Oliyad Husen, Habtemu Jarso, Lenco Bati

**Affiliations:** ^1^ Department of Medical Laboratory Science Institute of Health Bule Hora University, Bule Hora, Ethiopia; ^2^ Department of Public Health College of Health Science Madda Walabu University Shashemene Campus, Shashemene, Ethiopia; ^3^ Department of Pharmacy Institute of Health Bule Hora University, Bule Hora, Ethiopia

**Keywords:** Bule Hora town, Ethiopia, intensity, kindergarten children, prevalence, soil-transmitted helminths

## Abstract

**Background:** Soil-transmitted helminth (STH) infections are a common problem in Ethiopia. This parasite affects the physical and mental development of children, causing malnutrition and iron deficiency anaemia. However, there are inadequate studies that demonstrate the extent of STHs and associated factors among kindergarten children in South Ethiopia, particularly in the study area.

**Objective:** The aim of this study is to determine the prevalence, intensity, and associated factors of STH infection among kindergarten students in Bule Hora town.

**Methods:** An institution-based cross-sectional study was conducted among randomly selected 235 kindergarten children in Bule Hora town from January to February 2023. A pretested questionnaire was used to collect information on associated factors. EpiData and SPSS were used for data entry and analysis, respectively. Binary logistic regression analyses were performed to identify risk factors.

**Results:** In the present study, the prevalence of STH was 28.5% (95% CI: 22.7%–34.3%). *A. lumbricoides* 26 (11.1%) was the most common parasite identified. The arithmetic mean (+SD) of the egg counts for each species of STHs was as follows: *A. Lumbricoides* 1886.9 (+2103.2), *T. Trichiura* 299.2 (+305.8), and hookworms 756.1 (+739.4). Factors that showed significant association in the current study were hand washing after the toilet (AOR: 2.992, 95% CI: 1.299–6.891,), fingernail trimming (AOR: 3.646, 95% CI: 1.704–7.798,), and shoe wearing habit (AOR: 2.143, 95% CI: 1.170–3.924,).

**Conclusion:** STH infection is a health problem among kindergarten children in Bule Hora town. Comprehensive health education on the value of hygienic habits, improved sanitation, and routine deworming of kindergarten children should be included in prevention and control efforts.

## 1. Introduction

Soil-transmitted helminth (STH) infections are highly prevalent in regions with limited resources [1]. STH infections occur when intestinal parasites that grow in soil are transmitted to humans through soil contaminated with feces [2]. Currently, the most common species of STHs that cause soil-transmitted helminthiases are *Ascaris lumbricoides*, *Trichuris trichiura*, and hookworms (*Ancylostoma duodenale* and *Necator americanus*) [3]. Preschool children attending daycare centers are particularly vulnerable to STH infection [4].

STH infections are considered to be a major public health problem in many resource-limited countries, in contrast to developed countries where effective control, urbanization, and other socioeconomic factors have created better conditions for reducing the prevalence of STH infections [5]. The estimated number of people known to be infected with *A. lumbricoides, T. trichiura*, and hookworm worldwide was 820, 440, and 460 million, respectively, according to the 2017 WHO report [6]. STH infections are a major public health problem in Ethiopia [1]. Poor hygiene, low immune status, overcrowding, close contact with soil, lack of latrines, and inadequate water supply in schools are some of the factors that put school-aged children at high risk of soil-transmitted infections [7–9]. Food, drink, soil, and untrimmed (dirty) fingernails are just a few examples of fecal-contaminated objects that can directly or indirectly spread these parasites. Increased feco-oral transmission has been identified in several studies as a potential source of exposure to parasitic diseases [10, 11].

Prevention and control of STH is based on avoiding fecal contamination of food and water [5]. Studies have shown that frequent hand washing, wearing shoes, and avoiding eating soil are among the most effective methods of preventing STH infection [12, 13].

Health promotion and education to improve personal hygiene and encourage hand washing with soap and water, proper latrine use and food handling also play an important role in reducing person-to-person transmission [14]. In addition, community-based mass deworming programs, especially among preschool children, through the annual or biannual distribution of a single dose of a broad-spectrum antihelminthic drug is the currently advocated and practiced control strategy [15].

Children and adolescents are the most commonly infected with intestinal parasites [16, 17]. Unfortunately, children are more likely to suffer from severe illness and intestinal parasitic diseases [18]. Children in nursery schools face several worrying risks from STH. Children's survival, appetite, growth and physical fitness, school attendance, and cognitive function are negatively affected, as are their school attendance and cognitive function [19].

Regular screening for STH may be important to reduce the range of adverse health effects that these infections can cause. Previous studies in Ethiopia have focused on the distribution of different intestinal parasites in different study groups [20]. However, the prevalence of STH and associated factors among kindergarten children in South Ethiopia, including the current study area, has not been adequately addressed. Consequently, this research sought to determine the prevalence, intensity, and contributing factors of STHs among kindergarten children in Bule Hora town, Oromia region, Southern Ethiopia.

## 2. Materials and Methods

### 2.1. Study Area and Period

A study was conducted from February to March 2023 among selected students of kindergarten (KG) schools in the Bule Hora town, Oromia region. Bule hora town is the administrative center of West Guji Zone and is located 467 km away from Addis Ababa in southern Ethiopia. In 2007, the Central Statistical Agency of Ethiopia (CSA) conducted a census in the town and found a total population of 27,820 people, comprising 14,519 males and 13,301 females. The study was conducted in both government and private schools, and there are 15 nursery schools in the town.

### 2.2. Study Design

A cross-sectional study was conducted among KG students to assess the prevalence and intensity of STHs and their associated factors in Bule Hora town.

### 2.3. Source Population

All the children who were found in Bule Hora KG School.

### 2.4. Study Population

The study included all selected KG school children from student registration.

### 2.5. Inclusion and Exclusion Criteria

The study included all randomly selected kindergarten children in the selected schools, who agreed to provide all required data and stool samples. The study did not include children who did not voluntarily provide stool samples and all children who had taken antihelminthic medication 2 weeks before screening.

### 2.6. Sample Size

The sample size was determined based on the assumption that the proportion of STHs in kindergarten children is 16.7%, which was derived from a report of a study conducted in 2021 [21]. The resulting sample size was 214, based on a 95% confidence level (*Z* = 1.96) and a 5% margin of error (*d*).

 $$\begin{array}{c} n={\left(Z_{\alpha /2}\right)}^{2}\times \frac{p\left(1-p\right)}{d^{2}}={\left(1.96\right)}^{2}\times \frac{0.167\left(1-0.167\right)}{{\left(0.05\right)}^{2}}=214 \end{array}$$

A 10% margin of error was added to the calculated sample size, resulting in a sample size of 235 to minimize the error due to the likely occurrence of nonresponse.

### 2.7. Sampling Technique

Four private kindergartens and one public kindergarten were selected in proportion to the total number of students. During the study period, a total of 2049 students were enrolled in the selected kindergartens. The selected KG schools were Abbayi (*N* = 102), B/shifoo (*N* = 168), B/maddaa (*N* = 143), Siinayee (*N* = 980) and Q/maariyaam (*N* = 656). A sample was then drawn from the enrollment registers of each kindergarten. The stratification of the KG students was done considering the different KG schools selected. The complete required sample was then selected using a systematic random sampling technique after proportionally distributing the number of research participants in each class.

### 2.8. Data Collection and Laboratory Processing

Two data collectors used a face-to-face interview procedure with a pretested structured questionnaire to collect all essential background information from the guardians or family of the children. The pretest examination took place at the chosen kindergarten school (Walif) in the town of Fincawa. After appropriate instruction, each study participant was given a fecal cup, applicator stick, and soft tissue paper (for cleaning) to provide 2 g of their fresh fecal sample. Each sample was then labeled and delivered to the Bule Hora University Teaching Hospital within 30 min along with a completed questionnaire for processing and analysis. Two experienced laboratory technicians analyzed the stool samples using the Kato–Katz concentration method to count and look for STH eggs. Both data collection and microscopic examination of the parasite were regularly supervised. Stool samples were processed according to the standard procedure of the Kato–Katz concentration technique for microscopic examination [22] on the same day that the participants provided their stool samples.

Two Kato–Katz slides were prepared for each sample, and the slides were examined for hookworms at a nearby health facility within 45 min. The average number of eggs on both slides was multiplied by 24 to obtain the number of eggs per gram of feces for hookworm, *Ascaris lumbricoid*, and *Trichuris trichiura* parasites. The intensity of infection was categorized as mild, moderate, or severe based on the fecal egg count for each STH identified; for *A. lumbricoidesis*, mild infection (1–4999 epg), moderate (5000–9999 epg), and severe (greater than 10,000 epg); for *T. trichiura*, mild (1–999 epg), moderate (1000–9999 epg), and severe (greater than 10,000 epg). The World Health Organization (WHO) classification of hookworm is mild (1–1999 epg), moderate (2000–3999 epg), and severe (greater than 4000 epg) [23].

### 2.9. Quality Assurance

The questionnaire was first prepared in English and then translated into the local language (Amharic and Afaan Oromo) by language experts. The questionnaires were regularly checked for completeness of the required data. Data collectors were trained before data collection. Samples were properly prepared and examined. Data were checked daily by the principal investigator and supervisor for completeness and accuracy of data collection. The laboratory result record form was properly retained for verification.

### 2.10. Data Analysis

Data entry and analysis were performed using EpiData version 4.6 and SPSS Windows version 26, respectively. The collected data were summarized using descriptive statistical methods. Infection intensity was determined for *A. lumbricoides*, *T. trichuria*, and hookworms and expressed as ova per gram (opg) of feces for each student. The influence of factors on the likelihood of STH was assessed using logistic regression analysis. A *p* value < 0.05 with 95% CI was considered statistically significant.

## 3. Results

### 3.1. Sociodemographic Characteristics

A total of 235 preschool children participated in the study, with a complete participation rate of 100%. The majority of the participants were either 6 or 7 years old. In terms of family size, most households (50.6%) had between three and five children. Regarding the educational level of mothers, the largest group (32.3%) had completed primary school. Furthermore, the predominant occupation of the families (46.8%) was agriculture (Table 1).

### 3.2. Hygiene-Related Factors

Regarding the hygiene status of the kindergarten students, most study participants (73.6%) do not have the habit of eating soil. Most of the kindergarten children (76.2%) wash their hands after using the toilet. The majority of kindergarten children (69.4%) cut their nails during the data collection period. Regarding the consumption of raw vegetables or fruits, most of the kindergarten children (60.4%) do not consume them (Table 2).

### 3.3. Prevalence and Intensity of STHs

**Figure 1:**
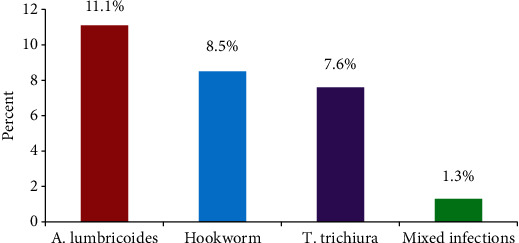
Distribution of soil-transmitted helminths in kindergarten students in the town of Bule Hora, February 1–31 March 2023.

A total of 79 of the 235 stool samples tested positive for at least one intestinal parasite, giving a prevalence of 33.6% (95% CI: 27.6%–39.7%). *A. lumbricoides*, 26 (11.1%), and hookworm, 20 (8.5%), were the most common parasites. The prevalence of STHs in this study was 28.5% (95% CI: 22.7%–34.3%). Of the 67 students with STH infections, 64 (95.5%) had one STH, and 3 (4.5%) had a double infection (*A. lumbricoides* and hookworm) (Figure 1).

Only one of the 29 KG students infected with *A. lumbricoides* had a severe infection, while the other 23 and 5 had mild and moderate infections, respectively. Nineteen of the 23 KG children with hookworm infection had a mild infection, while 4 had a moderate infection. Similarly, of the 18 KG children with *T. trichiura* infection, 15 had a mild infection, and 3 had a moderate infection. For each species of STH, the arithmetic mean (+SD) number of eggs was as follows: *A. Lumbricoides* 1886.9 (±2103.2), *T. Trichiura* 299.2 (±305.8), and hookworms 756.1 (±739.4).

### 3.4. Factors Associated With Prevalence of STHs

All variables were examined in the bivariate analysis, and those with a significance level of less than 0.25 were included in the multivariable analysis. The multivariable analysis used a backward logistic regression approach to assess the relationships between different factors. The study found that washing hands with soap after using the toilet, cutting fingernails, and the habit of wearing shoes were significantly associated with the prevalence of STHs among kindergarten children. Compared to KG students who washed their hands after using the toilet, those who did not wash their hands had a 3-fold (AOR: 2.992, 95% CI: 1.299–6.891) higher risk of contracting STH. Compared to KG students who cut their fingernails, those who did not have a 3-fold (AOR: 3.646, 95% CI: 1.704–7.798) increased risk of STH infection. KG students who did not wear shoes had a 2-fold (AOR: 2.143, 95% CI: 1.170–3.924) higher risk of STH than those who wore shoes (Table 3).

## 4. Discussion

Studies showing the prevalence and intensity of STH in different regions are essential to determine the population at risk of parasitic disease and to develop effective prevention and control strategies. In this study, the prevalence of STHs was 28.5% (95% CI: 22.7%–34.3%). This finding is similar to a study in Arbaminch (27.7%) [24] and Western Uganda (26.5%) [25]. This finding was lower than the results of similar studies conducted in Jimma (46.6%) [26], Nigeria (30.0%) [27], and Northwest Ethiopia (32.3%) [28]. This may be due to differences in study duration, environmental sanitation, and water supply. On the other hand, our finding was higher than other findings in Gonder town (13.8%) [21], Wanji (8.3%) [29], Ambo town (12.6%) [30], Kenya (12.9%) [31], South Africa (21.1%) [32], and Gurage zone (9.5%) [20]. Differences in the prevalence of STHs between studies may be explained by differences in geography, socioeconomic level of households, application of prevention and control strategies, and laboratory techniques used.

*A. lumbricoides* (11.1%) was the predominant parasite identified in the present study. This finding is consistent with studies in Gonder town [21] and northwestern Ethiopia [33]. This dominance of *A. lumbricoides* could be due to the wide distribution of the parasite and the large number of eggs laid by the fertile female parasite, which also contributes to the wide distribution of the parasite, and the robustness and resistance of the eggs, which enables them to survive unfavorable conditions. At a temperature of 5°C–10°C, eggs can withstand desiccation for 2–3 weeks, survive without oxygen for 2 years, and remain viable for 2 years [21, 34].

In terms of intensity, the majority of STH-positive children were infected with mild infection, while five children were infected with moderate infection. Only one child had a severe infection. However, another similar study in Gonder town [21] reported that almost all children infected with STH were infected with mild infection, and only one child was infected with moderate infection. The discrepancy between studies may be due to differences in the socioeconomic level of households, water supply, environmental sanitation, and geographical distribution.

Factors that showed a significant association in the current study were washing hands after going to the toilet (AOR: 2.992, 95% CI: 1.299–6.891), cutting fingernails (AOR: 3.646, 95% CI: 1.704–7.798), and wearing shoes (AOR: 2.143, 95% CI: 1.170–3.924). Children who did not wash their hands after using the toilet were almost three times more likely to be infected with STH. Washing hands after using the toilet is very important to prevent the parasite from getting into children's food. In addition, children often put their hands in their mouths, which increases the risk of contracting the parasite if they do not wash their hands after using the toilet. Children who did not cut their fingernails were 3.65 times more likely to be infected with STH than those who cut their fingernails. Children with untrimmed fingernails are at risk of picking up parasites when using the toilet or handling contaminated soil, exposing them to STH disease. Children who did not wear shoes were at least twice as likely to contract STH. Wearing shoes is important to reduce hookworm infection, which is transmitted by filariform larvae found in soil. Children consistently fail to wear shoes given to them by their parents. However, previous studies have identified age, kindergarten level, mother's education, mother's occupation, and father's education as variables significantly associated with STHs [21].

The current study has some limitations. It is cross-sectional and does not distinguish between cause and effect. It is also not a community-based study, which makes it difficult to conclude the community.

## 5. Conclusion

The prevalence of STH infections among kindergarten children is high in Bule Hora town. Hand washing after defecation, fingernail trimming, and shoe-wearing habits were factors significantly associated with the prevalence of STH among kindergarten children in the study area. Comprehensive health education on the value of hygienic habits, improved sanitation, and routine deworming of kindergarten children should be included in prevention and control efforts to reduce the prevalence of STH infections.

## Figures and Tables

**Table 1:** Sociodemographic characteristics of the study participants in the town of Bule Hora, February 1–March 31, 2023.

**Variables**	**Category**	**Frequency**	**Percent (100%)**
Age of KG students in years	4–5	92	39.1
	6–7	143	60.9

Sex of participants	Male	111	47.2
	Female	124	52.8

Family size of respondents	1–2	21	8.9
	3–5	119	50.6
	6–9	73	31.1
	≥10	22	9.4

Educational status of parents	Cannot read and write	69	29.4
	Elementary	76	32.3
	Secondary	59	25.1
	Above secondary	31	13.2

Occupation of parents	Farmer	110	46.8
	Merchant	36	15.3
	Employee	40	17.0
	Housewife	39	16.6
	Daily laborer	10	4.3

**Table 2:** Hygiene-related factors of study participants in the case of KG students in Bule Hora town, February 1–31 March 2023.

**Variables**	**Category**	**Frequency**	**Percent (100%)**
Soil eating habits	Yes	62	26.4
	No	173	73.6

Washing hands after toilet	Yes	56	23.8
	No	179	76.2

Fingernail trimming	Yes	72	30.6
	No	163	69.4

Habit of eating raw vegetables	Yes	93	39.6
	No	132	60.4

**Table 3:** Factors associated with the prevalence of soil-transmitted helminths among kindergarten students in Bule Hora town.

**Variables**	**Category**	**STHs (+) ** **N** ** (%)**	**STHs (−) ** **N** ** (%)**	**Total (** **N** **)**	**COR (95% CI)**	**AOR (95% CI)**	**p** ** value**
Sex	Male	35 (31.5)	76 (68.5)	111	1.324 (0.751–2.335)		0.28
	Female	32 (25.8)	92 (74.2)	124	1		

Age	4–5	29 (31.5)	63 (68.5)	92	1.272 (0.715–2.261)		0.995
	6–7	38 (26.6)	105 (73.4)	143	1		

Family educational status	No formal education	26 (37.7)	43 (62.3)	69	1.738 (0.679–4.452)	1.562 (0.572-4.268)	0.384
	Primary school	23 (30.3)	53 (69.7)	76	1.248 (0.487–3.199)		0.704
	Secondary	10 (16.9)	49 (83.1)	59	0.587 (0.205–1.682)		
	College and above	8 (25.8)	23 (74.2)	31	1		

Residence	Urban	35 (27.6)	92 (72.4)	127	1		
	Rural	32 (29.6)	76 (70.4)	108	1.107 (0.627–1.952)		0.78

Family occupation	Employed	15 (25.0)	45 (75.0)	60	1		
	Unemployed	52 (29.7)	123 (70.3)	175	1.268 (0.650–2.474)		0.199

Family size	1–2	5 (27.8)	13 (72.2)	18	1		
	3–4	40 (32.8)	82 (67.2)	122	1.268 (0.423–3.804)		0.73
	6–9	17 (23.3)	56 (76.7)	73	0.789 (0.246–2.532)		
	≥10	5 (22.7)	17 (77.3)	22	0.765 (0.182–3.210)		

Soil eating habits	Yes	17 (27.4)	45 (72.6)	62	0.929 (0.486–1.776)		0.97
	No	50 (28.9)	123 (71.1)	173	1		

Washing hands after latrine	Yes	8 (14.3)	48 (85.7)	56	1	1	
	No	120 (67.0)	59 (33.0)	179	2.950 (1.311–6.636)	2.992 (1.299-6.891)	0.010^∗^

Fingernail trimming habit	Yes	10 (13.9)	62 (86.1)	72	1	1	
	No	57 (35.0)	106 (65.0)	163	3.334 (1.588–6.998)	3.646 (1.704-7.798)	0.001^∗^

Shoe wearing habits	Yes	32 (22.1)	113 (77.9)	145	1	1	
	No	35 (38.9)	55 (61.1)	90	2.247 (1.261–4.004)	2.143 (1.170-3.924)	0.014^∗^

Habit of eating raw vegetables	Yes	23 (24.7)	70 (75.3)	93	1		
	No	44 (31.0)	98 (69.0)	132	1.366 (0.757–2.466)		0.095

Abbreviations: AOR, adjusted odd ratio; CI, confidence interval; COR, crude odd ratio.

^∗^Statistical significance at *p* < 0.05.

## Data Availability

The manuscript contained all the necessary data sets on which the conclusions of this work are based.
